# The Clinical Utility of Circulating HPV DNA Biomarker in Oropharyngeal, Cervical, Anal, and Skin HPV-Related Cancers: A Review

**DOI:** 10.3390/pathogens12070908

**Published:** 2023-07-05

**Authors:** Ioana Maria Andrioaie, Ionut Luchian, Costin Dămian, Giorgio Nichitean, Elena Porumb Andrese, Theodor Florin Pantilimonescu, Bogdan Trandabăț, Liviu Jany Prisacariu, Dana Gabriela Budală, Daniela Cristina Dimitriu, Luminita Smaranda Iancu, Ramona Gabriela Ursu

**Affiliations:** 1 Department and Preventive Medicine and Interdisciplinarity (IX)—Microbiology, Faculty of Medicine, “Grigore, T. Popa” University of Medicine and Pharmacy, 700115 Iasi, Romaniacostin-damian@umfiasi.ro (C.D.); luminita.iancu@umfiasi.ro (L.S.I.); ramona.ursu@umfiasi.ro (R.G.U.); 2 Department of Periodontology, Faculty of Dental Medicine, “Grigore T. Popa” University of Medicine and Pharmacy, 700115 Iasi, Romania; 3 Department of Medical Specialties (III)—Dermatology, Faculty of Medicine, “Grigore T. Popa” University of Medicine and Pharmacy, 700115 Iasi, Romania; 4 Department of Morpho-Functional Sciences II—Physiology, “Grigore T. Popa” University of Medicine and Pharmacy, 700115 Iasi, Romania; 5 Department of Ortopedy—Traumatology, 700115 Iasi, Romania; 6 Clinical Hospital of Infectious Diseases “Sf. Parascheva”, 700116 Iasi, Romania; 7 Department of Removable Dentures, Faculty of Dental Medicine, “Grigore T. Popa” University of Medicine and Pharmacy, 700115 Iasi, Romania; 8 Department of Biochemistry, “Grigore T. Popa” University of Medicine and Pharmacy, 700115 Iasi, Romania; 9 Gynecology and Obstetrics Hospital-Cuza Voda, 700038 Iasi, Romania

**Keywords:** biomarker, HPV, ctDNA, early detection, relapse

## Abstract

Human papillomavirus (HPV) is recognized as being related to a wide variety of known cancers: cervical, oropharyngeal, anal, vaginal, penile, and skin. For some of these cancers, rigorous algorithms for screening, therapeutical interventions, and follow-up procedures have been established. Vaccination using the nonvalent anti-HPV vaccine, which prevents infection regarding the most frequently involved high-risk HPV types (16, 18, 31, 33, 45, 52, and 58) and low-risk HPV types (6 and 11), has also extensively prevented, controlled, and even eradicated HPV infections. Still, even with all of these multidisciplinary interventions, the burden of HPV cancers is still high worldwide. The circulating DNA of HPV-induced cancers is thought to be an adequate biomarker for optimizing the control of these virus-related cancers. We analyzed the literature published in the last 5 years regarding ctDNA and four of the above-mentioned cancers. The most frequently used assay for ctDNA detection was the droplet digital PCR assay, used for the management of therapy in the late stages of cancer. ctDNA could not be used for early detection in any of the studied cancers. The OPSCCs were the most frequent cancers analyzed via ctDNA assays. Larger, properly designed cohort studies might establish the clinical utility of this biomarker.

## 1. Introduction

The burden of cancer cases attributable to HPV infection is very high, as this infection causes almost 5% of all new cancer cases worldwide. Annually, in women, HPV infections cause 530,000 cancer cases located in the cervix, 18,000 in the anus, 8500 in the vulva, 12,000 in the vagina, and 5500 in the oropharynx. In men, HPV infections cause 17,000 cases of anal cancer, 13,000 cases of penile cancer, and 24,000 cases of oropharyngeal cancer annually [1]. HPV-associated cancers of the head and neck are more commonly diagnosed in high-income countries, and genital warts have decreased significantly since the HPV vaccine was introduced [2].

The natural history of HPV-related cancers remains partially understood, with cervical cancer being the most analyzed HPV-related cancer. Recently, in 2022, the International Agency for Research on Cancer (IARC) published a handbook on cervical cancer screening, which presents and summarizes the published evidence on screening methods and practice through narrative and systematic reviews, as well as quality assessments. Additionally, it describes the natural history of cervical cancer, from a normal cervix to HPV infection, the progression to pre-cancerous squamous lesions, and an invasion leading to squamous cell carcinoma (SCC) [3]. The major steps in the development of cervical cancer are characterized by productive infection, which involves transitory infections with the possibility of achieving clearance, and transforming infections, in which pre-cancerous lesions evolve into cancer without the possibility of regression [4].

The relative importance of carcinogenic human papillomavirus (HPV) types has been established by IARC groups; HPV 16 is uniquely carcinogenic, with a 62.4% attributable etiological fraction (the percentage of cancer caused by HPV 16), followed by HPV 18 and HPV 45, which are relatively important for cancers, especially adenocarcinomas when compared to precancers, with attributable etiological fractions of 15.3% and 4.8%, respectively. For clinical use, commercial HPV screening assays often detect a pool of carcinogenic (or high-risk) HPV types; the 14 types most commonly included in current HPV tests are HPV 16, HPV 18, HPV 45, HPV 33, HPV 58, HPV 31, HPV 52, HPV 35, HPV 59, HPV 39, HPV 68, HPV 51, and HPV 56 [3].

The natural history of HPV, from oral HPV infection to HPV-related oropharyngeal cancer, is not as well understood as cervical cancer, with some data remaining unknown. There are, however, accepted risk factors: oral sex, being a male, and being a current smoker; these are factors that favor the transition from HPV exposure to oral HPV infection. Regarding persistence risk factors, we can add older age and immunosuppression to those previously mentioned. The signs of HPV can appear within 1–2 years, and progression to oropharyngeal cancer can take up to several decades, although no reliable precursors or progression risks have been identified yet. There is a great need in the field for large natural history studies in diverse populations to characterize the steps from the acquisition of oral HPV infection to its progression into HPV-positive oropharyngeal cancer [5].

The natural history of HPV in men became more well-known after the HIM study of Giuliano AR et al. was published in The Lancet [6]. We found that the prevalence of any HPV type in men was highest in the genital area (50%), followed by HPV with anal localization (12%) and HPV with oropharyngeal localization (4%). The prevalence of HPV-induced cancers in males may change over time [1], depending on the size of the study group analyzed, the location of the study, the different sexual practices of those who were selected to take part in the research, and more importantly, the HPV vaccination status in the tested patients. Interestingly, the genital HPV prevalence was high among all age groups, being over 50% over a range of age groups from 18–19 to 45–70 years old, with a peak of 70% for the 30–34 age group. HPV prevalence is known to be higher in men who have sex with men (MSM) compared to men who have sex with women (MSW) for all HPV groups: any HPV type, oncogenic type, and HPV 16 type only. Anal canal HPV prevalence differs according to sexual behavior, being higher in MSM (47.2%) than in MSW (12.2%) [7]. Despite the high burden of HPV in the anus, especially in populations that are at the greatest risk of anal cancer, this is still a rare disease. It is possible that the anal canal may be less susceptible to malignant transformation than the cervix, even in spite of persistent oncogenic HPV infection [8].

Skin cancer is thought to be etiologically connected to cutaneous beta HPV types, especially non-melanoma skin cancer. By using transgenic mice, Viarisio D et al. demonstrated that beta HPV 38 E6 and E7 oncoproteins act as promoter and progression factors in multi-stage skin carcinogenesis [9]. A review mentions the plausible hypothesis that beta HPV types have evolved strategies to maintain the proliferative status of infected cells even if they have been damaged by UV radiation. In this way, infected cells have an increased probability of progressing to malignancy [10].

The above data underline the high burden of HPV-related infections and cancers and the fact that these represent an important health problem worldwide. Screening and vaccination are important interventions that could lead to a major reduction in HPV-related diseases. Given the fact that so many anatomically different regions can be affected, it is difficult to obtain an ideal assay to screen for all the above-mentioned cancers. Still, in recent years, many research groups worldwide have tried to analyze the suitability of ctDNA detection techniques for different HPV-related cancers.

In 2010, Pantel K et al. presented a clinical application: circulating tumor cells in the peripheral blood and disseminated tumor cells. The authors of this paper mentioned the possibility of detecting these markers with ultrasensitive methods for the prognosis of cancer patients, monitoring systemic anticancer therapies, and identifying therapeutic targets [11]. Since then, researchers from many different study groups have performed investigations regarding the possibility of using liquid biopsy approaches (which also include the detection of cancer-related biomolecules, such as cell-free DNA (cfDNA), miRNAs, and extracellular vesicles in the blood or plasma). cfDNA circulates in plasma as short DNA fragments emerging from very different origins, including viral DNA. Circulating tumor DNA (ctDNA) is released and spread by tumors as DNA fragments into the blood. These biomarkers could be used in early cancer detection, in the follow-up of detected diseases, and in the evaluation of responses and resistance to therapy [12]. These biomarkers have the advantage of being relatively non-invasive for patients [13].

Aim: in this narrative review, we aimed to investigate the clinical utility of ctDNA assays for HPV-related oropharyngeal, cervical, anal, and skin cancers.

## 2. Materials and Methods

**Figure 1:**
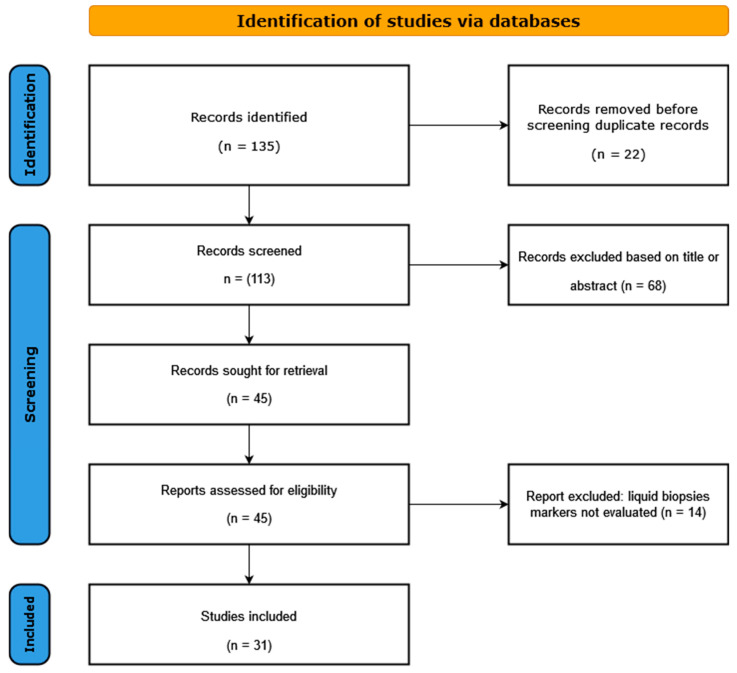
Flow diagram of selected studies in the present analysis.

Literature search and study selection: A systematic search of the PubMed and the EMBASE databases was carried out for all the published studies in the last 5 (2018–2023) years, using the following search algorithm: circulating DNA HPV AND cervical cancer, circulating DNA HPV AND oropharyngeal cancer, circulating DNA HPV AND anal cancer, and circulating DNA HPV AND skin cancer. Our systematic analysis identified 55 articles for oropharyngeal squamous cancer (OPSCC), 41 for cervical cancer (CC), 15 for anal cancer (AC), and 2 for skin cancer (SC). After a careful analysis of all these articles by two different team members, we excluded the review papers and only analyzed studies that were related to our research topic, giving 17 studies for OPSCC, 6 for CC, 7 for AC, and 1 study for SC (Figure 1). The above-mentioned 31 studies were included in the final analysis. The following criteria were used for the study eligibility assessment: (a) the study analyzed the viral DNA from a liquid biopsy obtained from human participants with HPV-related cancer; (b) original full-text articles; (c) sufficient data presented for the calculation of risk estimates; (d) detailed information provided on the design of the study.

## 3. Results

We identified 17 studies that evaluated ctDNA in oropharyngeal squamous cell carcinoma (OPSCC). A total of 12 of these studies were performed in the USA, two in Japan, and the rest in Europe. The main assay used for ctDNA HPV evaluation was digital droplet PCR (ddPCR) (*n* = 15), followed by real-time multiplex PCR (*n* = 2). Three papers used commercial ddPCR assays (e.g., circulating tumor tissue modified viral—TTMV), and the other authors used in-house optimized real-time PCR assays. Most studies collected blood and preserved plasma before and after the therapeutic intervention, which was either surgery alone or surgery with radio-chemotherapy (Table 1).

Two papers were clinical trial protocols for HPV-related OPSCC patients. One study used a NavDX assay (Naveris, Cambridge, MA) in its first prospective clinical trials, showing that quantitative cell-free HPV-DNA (cfHPV-DNA) levels could help to identify patient candidates for therapy de-escalation in HPV-related OPSCC [14]. The second study was a study protocol for a prospective study, a clinical trial, which intended to use quantitative cfHPV-DNA and ddPCR during induction, and (chemo)radiation during post-treatment follow-up. For these authors, quantitative cfHPV-DNA represents a promising, reliable, and convenient biomarker-driven strategy to guide personalized treatment in HPV-positive OPSCC [14,15].

The authors of the studies mentioned in Table 1 performed prospective, cross-sectional, retrospective clinical case series, transversal studies, and case presentations. The largest study is the one by Berger BM et al., 2022, Harvard Medical School, Boston, Massachusetts, in which 1076 consecutive patients were investigated [16]. Regarding the rest of the OPSCC-related studies below, the number of patients was below 200. The sensitivity values of the ctDNA assay, as reported by the authors, were between 56% and 100%, and the specificity values were between 88.9% and 100% (Table 1).

**Table 1:** HPV DNA biomarkers in the assessment of OPSCC.

Author, Year, Country	Study Design/ Ethnicity/ Sample Size, Type	Assay Type/ Parameters	Results	Clinical Utility
Li LQ et al., 2023, Edinburgh, UK, [17]	Prospective study Total of 86 patients following non-surgical treatment of OPSCC; European Blood samples before and after treatment	ddPCR HPV cell free DNA (cfDNA) PPV = 100% NPV = 71%	Overall, 8 patients with partial response and one patient with detectable HPV cell-free DNA at about 12 weeks after treatment.	In patients with a partial response, the detection of HPV cfDNA may be used to optimize target salvage treatment.
Rettig EM et al., 2022, Boston, MA, USA [18]	Cross-sectional study Total of 110 HPV-positive OPSCC patients; white (95%) Blood samples	Pretreatment circulating TTMV HPV DNA of 5 genotypes (16, 18, 31, 33, and 35) commercially available ddPCR assay Lower SE	Circulating TTMV HPV DNA was detected in 98 patients (89%). The most frequently detectable TTMV HPV DNA was genotype 16. Circulating TTMV HPV DNA and clinical N stage were the most strongly associated.	Circulating TTMV HPV DNA was statistically significantly associated with nodal disease at HPV-positive OPSCC diagnosis.
Berger BM et al., 2022, Harvard Medical School, Boston, MA, USA [16]	Retrospective clinical case series Total of 1076 consecutive HPV-driven OPSCC patients from across 108 U.S. sites Plasma samples	Circulating TTMV-HPV DNA tests (NavDx, Naveris Laboratories) Quantification of five high-risk HPV subtypes (16, 18, 31, 33, 35) PPV = 95% NPV = 95%	Circulating TTMV-HPV DNA was positive in 80 of 1076 (7.4%) patients, with follow-up available for all.	Circulating TTMV-HPV DNA testing has the potential to be applied in routine clinical practice.
Zarrabi KK et al., 2022, Philadelphia, PA, USA [19]	Case presentation A 78-year-old, male patient, 7 years post-treatment for squamous cell carcinoma in the lung (p16+ HNSCC)	HPV ctDNA	ctDNA can be considered a tool for longitudinal disease monitoring during therapy.	In HPV-mediated oropharyngeal carcinoma, ctDNA has the potential to be used in determining the response to treatment.
Routman DM et al., 2022, Rochester, MI, USA [20]	Prospective study Total of 45 samples pre-op; 159 samples post-op; HPV-positive OPSCC patients; American Serum samples	Multi-analyte PCR assay -	Detectable post-op ctHPVDNA, which is significantly associated with worse recurrence-free survival.	A future prospective study will investigate the association of detectable ctHPVDNA post-surgery with recurrence while also comparing it to established clinical and pathologic risk factors.
Warlow SJ et al., 2022, Edinburgh, UK, [21]	Prospective cohort Total of 104 HPV OPSCC patients; European Plasma	ddPCR assay cfDNA for five HPV types SE ˂ 100%	Persistently elevated cfDNA-HPV levels post-treatment were associated with treatment failure.	Pretreatment plasma cfDNA-HPV analysis had a high concordance with tissue-based assays. cfDNA-HPV ddPCR assay may have utility in the management of OPSCC.
O’Boyle CJ et al., 2022, Boston, MA, USA [22]	Prospective observational study Total of 33 HPV+ OPSCC patients; American Pre-and post-treatment plasma samples (days 1, 7, 30 post-op)	ddPCR assays for HPV genotypes 16, 18, 33, 35, and 45 SE = 89–98% SP = 97–100%	ct HPV DNA level rapidly decreased to <1 copy/mL by day 1 post-surgery in patients without pathologic risk factors for recurrence.	ctHPVDNA levels 1 day post-surgery are associated with a risk of residual disease in HPV+ OPSCC patients.
Cao Y et al., 2022, Ann Arbor, MI, USA [23]	Randomized trial Total of 34 patients with AJCC8 stage III OPSCC; American; serial blood samples	ctDNA values were analyzed for the prediction of freedom from progression, and correlations with aggressive tumor subvolumes with low blood volume, low apparent diffusion coefficient, and metabolic tumor volume. Plasma HPV 16/18 ctDNA analysis	Low pretreatment ctDNA and an early increase in ctDNA at week 2 compared with baseline were significantly associated with superior FFP.	Therapy response in stage III OPSCC may be predicted by analyzing early ctDNA kinetics during definitive chemoradiation.
Akashi K et al., 2022, Tokyo, Japan, [24]	Cohort, follow-up study Total of 25 p16-positive OPSCC patients; Asian Plasma samples	ctDNA ddPCR Real-time PCR, primers, and probe sets targeting the specific regions of E6 and E7 of the HPV viral DNA. SE = 56%	ct HPV DNA was detected in 14 (56%) of the 25 patients.	ct HPV DNA can be a prospective biomarker for predicting the recurrence of p16-positive OPSCC.
Tanaka H et al., 2022, Osaka, Japan, [25]	Transversal study Total of 45 HPV-related OPSCC patients; Asian Oral gargle samples Blood samples	ct HPV 16 DNA In-house ddPCR HPV 16 copy number/tumor genome SE = 93% SP = 97%	The sensitivity of ct HPV 16 DNA for HPV 16-related OPSCC was higher than that of oral biomarkers.	ctHPV16DNA is a potentially promising biomarker for HPV 16-related OPSCC.
Haring CT, et al., 2022, Ann Arbor, MI, USA, [26]	Longitudinal study Total of 12 patients; American Plasma samples	Analytically validated ddPCR assay for HPV 16 ctDNA ≥60% increase as a cut-off point for the prediction of progressive disease. SE = 88.9% SP = 88.9% PPV = 88.9% NPV = 88.9%	Treatment response is correlated with longitudinal changes in HPV 16 ctDNA levels. ctDNA responses are observed earlier than with conventional imaging.	HPV 16 ctDNA may be used to identify early signs of treatment failure, allowing the identification of patients that should be directed promptly toward clinical trials or alternative therapies.
Reder H, et al., 2020, Giessen, Germany, [27]	Pilot observational study Total of 50 HPV-driven OPSCC patients; European Plasma before therapy/during follow-up	cf HPV-DNA qRT PCR for DNA of HPV oncogenes E6, positive/negative SE = 83% SP = 95% E7, positive/negative: SE = 80% SP = 100%	Significantly reduced cfHPV-DNA levels after therapy for patients without clinical evidence of recurrence.	Plasma cfHPV-DNA detection correlates with the clinical course of disease in patients with HPV-driven OPSCC.
Veyer D et al., 2020, Paris, France, [28]	Prospective study Total of 66 p16+/HPV 16-positive OPSCC patients; European Plasma samples	HPV 16 ctDNA optimized ddPCR High SE High AC High RE	There was a significant association between HPV 16 ctDNA at baseline and the T/N/M status or OPSCC stage.	ddPCR HPV ctDNA assays could constitute a useful and non-invasive dynamic biomarker to select HPV-related OPSCC patients for potential treatment de-escalation and to monitor treatment response.
Rutkowski TW et al., 2020, Gliwice, Poland, [29]	Cohort study Total of 66 HPV-related OPC patients; European Blood samples	cfHPV16 DNA TaqMan-based qPCR SE = 100% SP = 98% PPV = 83% NPV = 100%	cfHPV16 DNA was found in five (28%) patients with an incomplete radiological response, while only in one (4%) with a complete radiological response.	cfHPV16 DNA may be used as a complementary biomarker to conventional imaging in patients with HPV-related OPC, and for the early identification of treatment failure.
Chera BS et al., 2020, Chapel Hill, NC, USA [30]	Cohort study Total of 115 nonmetastatic HPV-p16-positive OPSCC patients; American Plasma samples	Plasma ctHPVDNA, multianalyte ddPCR E7 genes encoded by high-risk HPV strain 16 and E7 gene for high-risk HPV strains 18, 31, 33, and 35 SE = 100% SP = 99% NPV = 100% PPV = 94%	A total of 28 patients had positive ctHPVDNA during post-treatment surveillance, 15 of whom were diagnosed with biopsy-proven recurrence.	ctHPVDNA detection in two consecutive plasma samples during post-treatment surveillance has high positive and negative predictive values for identifying disease recurrence in patients with HPV-associated OPSCC.
Chera BS et al., 2019, Chapel Hill, NC, USA [31]	Multi-institutional prospective biomarker trial Total of 103 p16-positive OPSCC patients; American Plasma samples	Optimized multianalyte ddPCR assays to quantify ctHPVDNA (types 16/18/31/33/35) SE = 89% SP = 97%	Pretreatment ctHPV16DNA copy number correlated with disease burden, tumor HPV copy number, and HPV integration status.	Rapid clearance of ctHPVDNA may predict the likelihood of disease control in HPV-associated OPSCC patients treated with definitive CRT and may be useful in selecting patients for therapy de-escalation.
Damerla RR et al., 2019, New York, NY, USA [32]	Cohort study Total of 97 HPV-positive OPSCC patients; American Plasma samples	HPV 16 ctDNA ddPCR SE = 92.8% SP = 100%	HPV 16 ctDNA was detected in all the patients with low-volume disease.	HPV 16 and HPV 33 ctDNA ddPCR could be used to detect small tumors, which indicates potential in early detection screening trials and in disease response monitoring.

Abbreviations: ddPCR—droplet digital PCR; ct DNA—circulating tumor DNA; cf DNA—cell-free DNA; SE—sensitivity; SP—specificity; PPV—positive predictive value; NPV—negative predictive value; AC—accuracy; RE—reproducibility.

We identified six studies that evaluated ctDNA in cervical carcinoma (CC). Three of these studies were performed in Europe, one in Canada, and one in Thailand. Five studies used ddPCR assays for ctDNA HPV evaluation, and the research group from IARC used the E7-MPG bead-based HPV genotyping assay, based on Luminex technology. All studies analyzed the blood from patients at different stages of HPV cervical infection (Table 2).

The authors of the below-mentioned studies performed cohort studies, case–control studies, and prospective multi-center studies. The number of CC cases analyzed for ct DNA HPV in these studies was between 23 and 206 cases. The sensitivity of the ctDNA assay was between 30.77 and 100.00%, and specificity was 100% in all the studies below.

We identified seven studies that evaluated ctDNA in anal carcinoma (AC). Two of these studies were performed in the USA, one in Brazil, and the rest were carried out in Europe. Five studies used a ddPCR assay for ctDNA HPV evaluation, three used NGS, one used in situ hybridization, and one used flow cytometry. Plasma samples were analyzed in all seven of these papers (Table 3).

The authors of the below-mentioned studies performed cohort studies, case–control studies, and prospective multi-center studies. The number of AC cases analyzed for ct DNA HPV in these studies was between 2 and 288 cases. The sensitivity of the ctDNA assay reached 100% for both sensitivity and specificity in detecting ctDNA in anal cancers.

Péré H et al., 2021, presented a very interesting case of a patient who died from HPV 16-induced high-grade anal intraepithelial SCC with vertebral HPV 16-positive metastasis. By using the Capture-HPV method and then next-generation sequencing (NGS), the authors found that HPV 16 was detected episomally in all tested samples: anal biopsy samples, plasma samples, and vertebral metastasis biopsy. The availability of the above-mentioned technologies could be used in future prospective studies to anticipate overall and progression-free survival and to offer patients optimized and personalized treatment [39].

**Table 3:** Liquid biopsy biomarkers in the assessment of anal cancer (AC).

Author, Year, Country	Study Design/ Ethnicity/ Sample Size, type	Assay Type/ Parameters	Results	Clinical Utility
Ruano APC et al., 2023, São Paulo, Brazil, [40]	Cohort study Total of 15 non-metastatic anal squamous cell carcinoma (SCC) patients; Brazilian Blood samples	Circulating tumor cells (CTCs) were isolated/quantified by ISET^®^ protein expressions, immunocytochemistry, HPV DNA, and chromogenic in situ hybridization.	CTCs were detected in all patients at baseline. HPV DNA was found in CTCs in 14/15 patients (93.33%) at baseline and in 7/9 (77.7%) after treatment. Three patients expressed ERCC1 in CTCs after treatment, one of which showed disease recurrence.	The detection of HPV in CTCs from patients with non-metastatic anal SCC is feasible and appears to be a sensitive diagnostic method.
Liauw SL et al., 2021, Chicago, IL, USA [41]	Two case presentations Anal SCC patients Peripheral blood samples	Circulating, tumor-tissue-modified HPV DNA (TTMV-HPV DNA) digital PCR PPV = 94% NPV = 100%	HPV genotyping confirmed the presence of HPV 16 DNA in all tested cases at diagnosis.	TTMV-HPV DNA levels, during patient follow-up after treatment, were correlated with disease status, including one case with progressive local recurrence within 2 months and one case with 12 months of local control, both confirmed by biopsy.
Lefèvre AC et al., 2021, Aarhus N, Denmark, [42]	Cohort study Total of 88 anal SCC patients; European Serial blood samples	In-house multiplex ddPCR for plasma HPV subtypes 16, 18, 31, 33, 51, 58 SE = 82% SP = 67%	Plasma HPV was detectable in 52/88 patients, and pretreatment levels correlated with tumor stages.	During CRT, plasma HPV can be used to divide patients with anal SCC into groups with significantly different risks of failure.
Lee JY et al., 2020, London, UK, [43]	Prospective study Total of 33 HPV-positive AC; European Plasma samples	Circulating HPV-DNA (cHPV DNA) novel next-generation sequencing (NGS) assay; panHPV-detect, eight high-risk HPV genomes (16, 18, 31, 33, 35, 45, 52, and 58) SE = 100% SP = 100% PPV = 100% NPV = 100%	pre-CRT samples, panHPV-detect: 100% sensitivity and specificity for HPV-associated AC PanHPV-detect was found in cHPV-DNA in 100% of patients with T1/T2N0 AC.	panHPV-detect is a highly sensitive and specific NSG assay for the identification of cHPV-DNA in plasma at diagnosis. Post-treatment cHPV-DNA levels may predict the clinical response to CRT.
Bernard-Tessier A et al., 2019, Paris, France, [44]	Cohort study Total of 59 AC patients; European Serum samples Pre-/post-CT	HPV 16 ctDNA quantification by ddPCR SE = 67% SP = 70% PPV = 80% NPV = 54%	HPV 16 ctDNA was detected in more than 90% of the baseline samples. Residual HPV 16 ctDNA was detected in 38.9% of patients, associated with shorter progression-free survival.	This prospective study shows that HPV ctDNA level pre-CT and HPV ctDNA negativity post-CT have a significant prognostic impact.
Damerla RR et al., 2019, New York, NY, USA, [32]	Cohort study Total of 8 HPV-positive AC; White Plasma samples	HPV 16 and HPV 33 ctDNA ddPCR SE = 92.8% SP = 100%	HPV 16 and HPV 33 ctDNA were detected with an overall sensitivity of 95.6%. Overall, 7/8 AC patients had detectable HPV 16 ctDNA.	HPV ctDNA assayed by ddPCR is highly sensitive and specific for identifying HPV 16 and HPV 33 associated with cancer.
Cabel L et al., 2018, Paris, France, [45]	Cohort study Total of 33 patients HPV 16- or HPV 18-positive locally advanced AC; European Blood samples pre-/post-CRT.	HPV 16 or HPV 18 ctDNA detection by ddPCR SE = 88%	HPV ctDNA detected pre-CRT in 29/33 patients with stages II-III AC, associated with tumor stage. Overall, 3/18 patients post-CRT had residual HPV ctDNA, which is associated with a poor outcome.	This is one of the first studies assessing the prognostic value of ctDNA after CRT in locally advanced AC.

Abbreviations: ddPCR—droplet digital PCR; ct DNA—circulating tumor DNA; cf DNA—cell-free DNA; SE—sensitivity; SP—specificity; PPV—positive predictive value; NPV—negative predictive value; AC—accuracy; RE—reproducibility.

For skin cancer, we identified only one paper published in the past 5 years regarding blood testing for HPV in relation to cutaneous cancer. Hampras SS et al. conducted a skin screening study by collecting blood, eyebrow hair, and skin swabs, which were analyzed for viral DNA using a bead-based multiplex PCR-Luminex assay. The authors found that skin infection with gamma HPV infections could increase the chances of cutaneous malignancy development by recruiting immunosuppressive lymphocytes into the skin [46].

## 4. Discussion

In this study, we have evaluated the clinical utility of ctDNA in HPV-related cancers. We found that the most frequently used assay for this biomarker evaluation was ddPCR, which was both optimized in-house and commercially developed, followed by NGS, real-time PCR, and in situ hybridization assays. This subject is of interest because other authors have tried to analyze the test parameters of the ddPCR assay [47,48,49], mainly for OPSCC and cervical cancer. Here, we analyzed all of the HPV-related cancers, including oropharyngeal cancer, cervical cancer, anal cancer, and skin cancer. Analyzing blood is very convenient in practical laboratory settings when compared to processing FFPE (formalin-fixed, paraffin-embedded) samples, fresh tumor samples/biopsies, and even cervical cells. This explains the large number of papers that analyzed (under many research scenarios) the utility of ctDNA. Still, the use of ddPCR has not yet been reported in studies from low-income countries, which have a higher prevalence of cervical cancer, perhaps due to the costs of such assays.

### 4.1. Discussion about OPSCC

The OPSCC was the most frequent type of HPV-related cancer, which was evaluated by ctDNA HPV in our study approach. One reason could be that, for OPSCC, even though it is known that performing oral sex is a risk factor for oral HPV infection, there are no recognized HPV-induced precancer lesions like there are for CIN2/3 or AIN2/3. Older age, male sex, current smoking, and immunosuppression could be considered risk factors for oral HPV persistence, but there is no recognized precursor from oral HPV persistence to oropharyngeal cancer [50]. Researchers are looking for an adequate biomarker for this HPV-related cancer. A recent study published in 2023 recognizes the need for follow-up studies in larger cohorts to clinically validate the use of post-treatment HPV cell-free DNA in the management of OPSCC [17]. Rettig EM et al. found that assay sensitivity for diagnostic purposes may be lower among individuals without cervical lymphadenopathy, as they had patients with undetectable levels of five genotypes (16, 18, 31, 33, and 35) of HPV DNA but had predominantly clinical-stage N0 disease. The authors suggest the need for further clinical investigations [18]. By using the commercial assay TTMV-HPV DNA tests (NavDx, Naveris Laboratories), authors from Harvard Medical School, Boston, suggested that this assay could be used for future clinical- and guideline-endorsed strategies for HPV-driven malignancy surveillance [16]. When analyzing OPSCC patients with the same commercial assay, Chai RL et al. considered that this evaluation method could be used to improve morbidity and quality of life and could be used as an introduction to important biomarkers in therapeutic decision-making [14]. Routman DM et al. detected ctHPVDNA before surgical intervention; a significant number of the pre-operatory positive patients were ctHPVDNA positive post-surgical intervention before starting adjuvant radiation therapy [20]. O’Boyle CJ et al. were even more precise in their study, and they found that ctHPVDNA levels 1 day after surgery are associated with the risk of residual disease in patients with HPV-positive OPSCC, and thus it could be used as a personalized biomarker for selecting adjuvant treatment in the near future [22]. Haring CT et al. stated that ctDNA could be used to enable the early identification of treatment failure, providing the opportunity to optimize therapy and guiding such cases toward clinical trials or alternative therapies [26]. After identifying that those patients without clinical evidence of recurrence had significantly reduced cfHPV-DNA concentrations after therapy, Reder H et al. suggested the need for extensive clinical investigations if cfHPV-DNA is detected during the follow-up of patients with HPV-driven OPSCC [27].

All the above studies show how many research scenarios have been developed by clinicians in collaboration with laboratory doctors, with the aim of therapy optimization for OPSCC patients. Over the years, researchers have been pre-occupied with finding the best selection of HPV-driven OPSCC cases, for example, the strict algorithm used in the HPV AHEAD studies, which combined DNA HPV testing with mRNA HPV analysis and p16 positivity [51,52,53,54,55]. This algorithm was very efficient in selecting HPV-driven cases but inconveniently used FFPE samples; these carry a known risk in laboratory practice, as they identify invalid samples due to the improper quality of the specimen. Other authors extended the research from developing markers to predict the outcome of OPSCC patients (e.g., CD8^+^ TIL counts, age, T-stage, and E2 expression) [56] by analyzing different gene expression (e.g., SPARC, psoriasin, type I collagen and galectin-1) between HPV-positive and HPV-negative TSCC (tonsillar squamous cell carcinoma) and BOTSCC (base of tongue squamous cell carcinoma) [57] or analyzing other cancers such as HPV-related uvula, soft palate, and hypopharyngeal cancers [58,59]. All these research efforts to screen for OPSCC still aim to address the fundamental principles of screening, which refer to the category of patients to include, the markers to analyze, the most adequate assays to use for screening, and, very importantly, the management of screen-positive individuals [60,61].

The specificity in the OPSCC studies that we analyzed varied between 88.9 and 100%. The detected sensitivity of ctDNA HPV for OPSCC was between 56 and 100%. The lowest sensitivity was identified for 25 OPSCC Asian patients who were p16-positive. The highest sensitivity was reported by the authors who tested American patients using a ddPCR assay. Generally, a single study evaluates over 1000 samples. Some analyzed studies had between 12 and 200 samples evaluated for ct DNA in OPSCC patients. For the period analyzed (2018–2023), the highest number of studies was found for OPSCC. This could be explained by the lack of a classical screening method, which is available for CCs. There is a need for the identification of an optimal biomarker for the early detection and monitoring of OPSCC HPV-related patients.

A recent meta-analysis, which included 10 studies with a total of 457 HPV-positive HNSCC, identified a pooled sensitivity and specificity of 0.65 (95% CI) to represent the diagnostic performance of cfHPV-DNA. Campo F et al. concluded that cfHPV-DNA could be used as a biomarker for monitoring treatment response during the clinical trials of de-escalation therapy [62].

Another review supports ctHPV assessment in the early detection of disease progression and at any stage of managing a patient with HPV-related OPSCC [63].

However, the diagnostic value of cfDNA as a biomarker, especially ct HPV DNA in OPSCC, is under clarification. As the vast majority of OPSCC studies are from the USA, ethnicity does seem to play a role, but rather the logistics and the experience of the researchers using ddPCR for ct DNA evaluation do not. Future studies on HPV-induced OPSCC cases should be cost-efficient and follow ethical study designs that could further advance the secondary prevention of HPV-induced OPSCC. It is important to identify pre-cancerous lesions and to optimally manage biomarker-positive patients and HPV-DNA-positive OPSCC patients.

### 4.2. Discussion about Cervical Carcinoma

The natural history of cervical carcinoma is very well known, starting from the normal cervix to pre-cancerous lesions and leading to carcinoma (NILM; ASCUS, LSIL; HSIL, CIS—carcinoma in situ) [3]. We identified six articles that used ctDNA assays in cervical carcinoma studies, which suggests that there are still aspects to be improved in the management of cervical cancer. Bønløkke S et al. used ddPCR for detecting plasma cell-free HPV DNA in patients with HPV 16- or HPV 18-positive cervical cancer. The authors decided that this biomarker could not be used for the early detection of such tumors but could be used for the monitoring of these patients after therapy in advanced cases, as quantitative evaluations showed significant correlations between cf HPV DNA level and stage, tumor score, and tumor size [33]. A similar conclusion was found by another team of researchers, who found that E6 antibodies were present, especially in the early tumor stages of CC, while HPV ctDNA was detected at more advanced tumor stages [34]. Another possible application of this biomarker is to assess subsequent relapses by detecting residual HPV ctDNA at the end of chemo-radiotherapy [36]. Han K et al. identified that the HPV DNA level at 3 months is more accurate than 3-month fluorodeoxyglucose positron emission tomography imaging in detecting residual disease [38].

Over the years, the screening of cervical cancer and pre-cancerous lesions has been optimized by very well-known groups of researchers [64,65,66,67,68]. In 2009, Meijer CJ et al. established the requirements for HPV tests in primary cervical screening and validation guidelines for candidate HPV assays (e.g., the sensitivity of the candidate test for ≥CIN2 should be at least 90% of the sensitivity of Hybrid Capture 2, and the specificity of the candidate test for ≥CIN 2 should be at least 98% of the specificity of Hybrid Capture 2) [64]. After more than 10 years of research, with several well-conducted studies on cervical cancer, the recent (2020) ESGO-EFC position paper of the European Society of Gynaecologic Oncology and the European Federation of Colposcopy mentions, again, the necessity of using a clinically validated test for the screening of high-risk HPV types (13 high-risk types: HPV 16, 18, 31, 33, 35, 39, 45, 51, 52, 56, 58, 59, and 68), as only a small proportion of tests fulfill the validation criteria [68]. Moreover, this position paper recommends that HPV-based screening should continue in the post-vaccination era, as certain authors have proven the necessity of screening for the HR HPV types, which are not included in the nonvalent HPV vaccine [69]. The quality assurance for HPV screening should also involve quality assurance systems, as well as internal quality control, external quality assessment, and staff competence [70,71].

The specificity in the analyzed CC studies reached 100% in two papers. The sensitivity of ctDNA from CC was low, between 30.77 and 74.7%. Some studies mentioned that they had ”high sensitivity”, without any precise specification. Only two studies tested more than 200 patients. The other studies had a very low number of CC patients tested for ctDNA (between 23–39 patients). One possible explanation for this low number of tested patients is that in developed countries, the CC prevalence has decreased thanks to well-implemented organized screenings. Therefore, it is more time-consuming to perform larger studies. On the other hand, in low-income countries, where the prevalence of CC is high, ctDNA-related technologies from body fluids are still in development and may not be feasible for application yet. The presence of HPV-ctDNA is associated with unfavorable outcomes in cervical neoplasia.

A recent meta-analysis identified the low sensitivity (0.36) and high specificity (0.96) of cHPV DNA as being diagnostic for cervical cancer. The authors concluded that along with further optimization of the analyses to achieve higher sensitivity, liquid biopsies are promising diagnostic and prognostic biomarkers for the detection of CC [72].

We should also be aware of the effects and harms of cervical cancer screening, which should be considered in accordance with the principle of evidence-based decision-making, which states that before implementing an intervention (e.g., plasma HPV ctDNA detection), the balance of clinical benefits, harms, and costs should be evaluated [3].

### 4.3. Discussion about Anal Cancer

The epidemiology of HPV infection in men can be described by the results of the HIM study conducted by Giuliano AR et al., which found that the age-specific prevalence of any genital HPV infection among men was detected to be high among all age groups: above 50% [6]. Anal canal HPV prevalence differs by sexual behavior, being higher in men having sex with men (MSM), 47.2%, compared to men having sex with women (MSW), 12.2% [73]. Among men, anal HPV prevalence varies substantially by HIV status and sexual orientation; anal HPV prevalence in men does not vary by age. In women, anal HPV prevalence differs by HIV status and cervical HPV infection or cervical cytopathology [74,75]. The natural progression of anal HPV infection to the anal cancer precursor, anal high-grade squamous intraepithelial lesion (HSIL), and then to cancer is very similar to that of cervical cancer. There is a need for high-quality evidence that treating anal HSIL reduces the risk of anal cancer. Useful data will be provided by the ANal Cancer/HSIL Outcomes Research (ANCHOR) study, which is focused on a group that is at the highest risk of anal cancer: men and women living with HIV, those over 35 years of age, and those with biopsy-proven anal HSIL [76].

Studies on the clinical application of ctDNA detection for anal cancer had similar designs to the research on OPSCC and cervical cancer. Some authors tried to use this marker to optimally monitor this category of patients, and they expressed the intention to conduct studies on larger cohorts to identify if there is any association between the screening markers of anal cancer and HPV [40]. Other researchers have already used ctDNA as a biomarker for anal cancer, and they intend to use it further for follow-up on anal cancer therapy [41]. Using a very sensitive assay for quantifying six HPV types (16, 18, 31, 33, 51, and 58), one research team was able to optimize clinical therapeutical decisions during radio- and chemotherapy for anal cancer [42]. Researchers from France used NGS for a comprehensive HPV 16 analysis in plasma and a vertebral metastasis biopsy from a patient with anal carcinoma. They found that HPV was episomal in each sample, and they mentioned that HPV status could be an important biomarker for managing aggressive anal cancer [39]. A team of researchers assayed ct HPV DNA using NGS to evaluate chemo-radiotherapy efficiency after 12 weeks, and they registered different outcomes (partial response, complete response, relapse). Regarding this study, the assay used, which identified eight high-risk HPV genomes (16, 18, 31, 33, 35, 45, 52, and 58), had 100% sensitivity and specificity in predicting patient response to chemo-radiotherapy [43]. Bernard-Tessier A et al. proposed that ct DNA could be considered an optimal biomarker for anal cancer if it had an acceptable cost and a short turnaround [44]. Another research team mentioned that ctDNA for HPV 16 and 33 could be used for the early detection of small tumors in screening trials and in follow-up post-treatment [32]. Cabel L et al. noted that high ctDNA levels after CRT are associated with very poor outcomes, as HPV ctDNA can be detected before chemo-radiotherapy and becomes undetectable during oncologic therapy [45].

The specificity of ct DNA HPV (AC-related) in the analyzed studies varied between 67 and 100%. The sensitivity had the same values but varied among different studies, between 67 and 100%. Lee JY et al., 2020, identified SE = 100%, SP = 100%, PPV = 100%, and NPV = 100% in a study on 33 HPV-positive AC by using a novel NGS assay. None of the analyzed studies reached 100 patients (limits: 2–88 patients) [32,40,41,42,43,44,45,46].

A recent meta-analysis identified a high sensitivity (0.95) and specificity (1.0) of cHPV DNA for the diagnosis of AC. Additionally, the authors considered that cHPV DNA was useful in the prediction of treatment response or progression-free survival in AC and in post-treatment detection [72]. A review performed by a researcher’s team from INSERM, France, sustained that HPV ctDNA usually becomes undetectable during oncologic therapy and that the post-radio-chemotherapy detection of HPV ctDNA is significantly associated with unfavorable evolution [77].

As it is currently not known which technique (ddPCR vs. NGS) is more sensitive, larger studies are required, including clinical trials, to assess the clinical utility of ct DNA HPV.

The proper diagnosis, treatment, and follow-up of HPV-induced anal cancer is a subject of interest, as there are many guidelines from different research groups [78,79,80] that underline different proposals for the optimal management of anal cancers.

### 4.4. Discussions Skin Cancer

Skin cancer development is initiated by ultraviolet (UV) radiation, together with beta HPV types, which augment UV-induced DNA mutations [10]. Bandolin L et al. recognized the role of beta-HPV in the oncogenesis of keratinocyte carcinomas, together with UV radiation [81]. The IARC research group performed a series of studies in which they investigated the role of cutaneous HPV types in skin carcinogenesis. The authors quantified viral DNA corresponding to 46 β-HPV types by using a multiplex genotyping Luminex-based assay [82]. Another study analyzed data on HPV seropositivity, HPV DNA in eyebrow hairs, and SCC tumors by using a similar Luminex-based technology, and the authors found that infection with beta HPV type 2 in eyebrow hair was associated with a reduced risk of subsequent SCC among HPV-DNA-positive SCCs [83].

Extraction methods for cell-free DNA and ctDNA from blood samples could be a logistical challenge for some laboratories. In the case of OPSCC studies, Akashi et al. collected a volume of 10 mL of blood, in addition to samples, during routine medical practice. The blood was processed via centrifugation using specific parameters. The cell-free DNA solution was extracted from 1 mL aliquots of plasma using the Maxwell^®^ RSC ccfDNA Plasma Kit (Promega, Madison, WI, USA) [24]. Tanaka et al. extracted cell-free DNA from 3 mL of cryopreserved plasma, and this was suspended in a volume of 100 μL using reagents from the QIAamp Circulating Nucleic Acid Kit (Qiagen) [25]. Haring et al. isolated cell-free DNA from a 2 mL aliquot of plasma using the QIAamp^®^ MiniElute^®^ ccfDNA Mini Kit (Qiagen, Germantown, MD, USA) and diluted it at a ratio of 1:40 according to the manufacturer’s instructions [26].

By using cervical cancer samples, Bønløkke et al. extracted ccfHPV DNA from 30 mL of full-blood samples collected from each participant in Cell-Free DNA BCT^®^ collection tubes (Streck, La Vista, NE, USA). These samples were stored at room temperature (6–37 °C) for up to 14 days before DNA extraction, as per the manufacturer’s instructions [33]. Jeannot et al. isolated cfDNA in duplicate from 200 μL serum samples using the QIAamp Min Elute Virus Spin Kit (Qiagen). Elution was performed in 25 μL of supplied elution buffer, and these eluates were pooled and stored at −20 °C until the HPV ctDNA analysis was performed [35].

Lefèvre et al., in their anal cancer study, used a Chemagic 360 robot (PerkinElmer, Waltham, MA, USA) and a 1304 cfDNA purification kit (PerkinElmer) to purify DNA from 4 mL of thawed plasma, to which 191 base pair (bp) spike-in DNA was added [42]. Another paper reported that circulating HPV DNA was extracted from 5 mL plasma samples using the QIAamp Circulating Nucleic Acid Kit (Qiagen), and the resulting DNA was eluted in 50 μL of AVE buffer and stored at −20 °C [43].

These selected methods show that there are already different commercially available kits for extracting ctDNA or cfDNA, which could favor, to some degree, the large applicability of this assay.

One major limitation of detecting HPV using ctDNA assays is that it cannot be used to monitor disease progression from low- and high-grade lesions and carcinomas, especially in ASCUS (atypical squamous cells of undetermined significance), LGSILs (low-grade squamous intraepithelial lesions), and HGSILs (high-grade squamous intraepithelial lesions). Moreover, due to the costs of these methods, it is unlikely that HPV ctDNA assays can be used on women from low-income countries. The same limitation can be mentioned for pre-cancerous anal lesions.

## 5. Conclusions

In this comprehensive analysis of HPV-related cancers, we have summarized the known natural histories for each of these types of cancer and the available guidelines for screening. We identified ddPCR as the most frequently used assay for detecting and quantifying specific, high-risk HPV types. Further clinical optimization of this assay is still needed in terms of sensitivity, specificity, and positive and negative predictive values. HPV-driven OPSCC was the most commonly studied type of cancer using the ddPCR methods.

None of the studies used ctDNA for the early detection of any of the analyzed HPV-induced cancers. The most useful clinical applications in the analyzed studies were to assay ctDNA as a biomarker before and after surgical intervention, to de-escalate CRT therapy, and to better optimize surgical interventions. Larger cohort studies are necessary to confirm the clinical utility of ctDNA in HPV-related cancers.

## Figures and Tables

**Table 2:** HPV DNA biomarkers in the assessment of CC.

Author, Year, Country	Study Design/ Ethnicity/ Sample Size, Type	Assay Type/ Parameters	Results	Clinical Utility
Bønløkke S et al., 2022, Aarhus, Denmark, [33]	Proof-of-concept study Study cohort Total of 181 patients with HPV 16, 18 derived—CC; European Plasma samples	ccfHPV ddPCR SE = 55.8% increased to SE = 61.6% by adding an additional primer pair and probe	ccfHPV DNA was detected in 19 late-stage patients (63.33%), three early-stage patients (10.00%), and none of the CIN3 patients or controls.	ccfHPV DNA may not be a useful marker for the early detection of CCs.
Galati L et al., 2022, Lyon, France, [34]	Research study: validation of an alternative to ddPCR for HPV 16 Total of 124 HPV 16-positive CC patients; European Plasma samples	The performance of the E7-MPG bead-based HPV genotyping assay (E7 type-specific multiplex genotyping assay [E7-MPG]) was compared with those of DNA detection ddPCR and the detection of HPV 16 E6 antibodies SE = 74.7% SP = 97.8%	The study validated an alternative to ddPCR for HPV 16 ctDNA detection, which uses a bead-based HPV genotyping assay that offers a potential cost-reduction for clinical management due to the multiplex capability of the test.	The HPV 16 ctDNA biomarker appeared to be highly specific and, to a lesser extent, sensitive for the detection of CC, especially for patients with an advanced tumor stage.
Jeannot E et al., 2021, Paris, France, [35]	Cohort study Total of 206 patients HPV 16- or HPV 18-related CCs; European Serum samples	ddPCR for circulating HPV *E7* gene (as a marker for residual disease) in comparison to HPV integration site and *PIK3CA* mutations High SE	HPV ctDNA was detected during pretreatment in 63% (59/94) of patients, and persistent HPV ctDNA predicted relapse.	HPV ctDNA detection is a useful biomarker to predict relapse in cervical cancer.
Cabel L et al., 2021, Institut Curie, Paris and Saint Cloud, France, [36]	Retrospective cohort Prospective cohort Total of 39 patients with HPV-positive locally advanced cervical cancer (LACC); European Blood and tumor samples	HPV-ctDNA detection genotype-specific ddPCR. HPV 31, HPV 33, HPV 35, HPV 45, HPV 52, HPV 58 and HP V73. High SP	HPV ctDNA was successfully detected in 69% of patients (*n* = 38/55) before CRT for LACC, and nine patients had a rare genotype.	One of the largest studies to report HPV-ctDNA detection pre-CRT and show clearance of HPV ctDNA at the end of therapy in most patients.
Rungkamoltip P et al., 2021, Bangkok, Thailand, [37]	Case–control study Total of 39 CC patients; 29 controls; Asian serum samples	ddPCR for HPV 16/18 E7 cfDNA SE = 30.77% SP = 100%	HPV 16/18 E7 cfDNA had 30.77% sensitivity and 100% specificity for CC.	HPV E7 cfDNA for minimally invasive CC monitoring has excellent specificity and moderate sensitivity.
Han K et al., 2018, Toronto, ON, Canada, [38]	Prospective multi-center study Total of 23 women with stage IB to IVA CC; White Plasma samples	Plasma HPV DNA detected serially by ddPCR SE = 100%	All patients had detectable plasma HPV DNA pretreatment. A total of six patients had detectable plasma HPV DNA post-CRT, and three patients had developed metastases at 3 months.	Detectable plasma HPV DNA at the end of CRT is associated with inferior progression-free survival and predates the clinical diagnosis of metastases.

Abbreviations: ddPCR—droplet digital PCR; ct DNA—circulating tumor DNA; cf DNA—cell-free DNA; SE—sensitivity; SP—specificity; PPV—positive predictive value; NPV—negative predictive value; AC—accuracy; RE—reproducibility.

## Data Availability

Data are contained within the article.
